# The Life-Long Lunatic

**Published:** 1858-01

**Authors:** 


### THE LIFE-LONG LUNATIC.

On Friday, May 29, 1857, the following statement was made in the British House of Commons, by Mr. Ellice:

In an unfrequented part of *Ross-shire*, *Scotland*, was found a house, four and a half feet high, with walls of turf, two and a half feet thick, thatched with heather, but admitting rain in several places. The room was nine feet by seven, with a dirt floor. There was no window and no fireplace. There was no furniture; but a bed of boards, two feet from the ground. On these boards some loose moss was scattered, and on this sat a man, wrapped in a piece of blanket, old and dirty, with two pieces of old bed-cover, all his clothing; these were washed once a fortnight, two pieces of blanketing supplying their places, meanwhile. To that bed this man had been chained for thirty years, and had never left it. The chain was two and a half feet long; one end around his ankle, the other attached to a staple in the bed. His food consisted of potatoes, oatmeal, herring, and molasses. He was sixty-eight years old, and had been insane forty years. He clips his beard with a pair of scissors, once or twice a month, and never washes, except his face, once or twice a year. His only attendant was an old woman, of seventy years, who lived in the other end of the miserable hut: the woman was his sister, almost as poor as himself. He was helpless. He could not walk. He had sat so long in bed, with his legs bent on his thighs, that the knees had become as inflexible as iron. For all these long thirty years, had these two, the brother and the sister, dwelt in misery together; and together might have rotted in the deepest depth of want and degradation, but for a person born in Boston, a school-teacher, of a constitution so frail, that she can scarcely walk half a mile at a time—an American woman, without "position," as the world views it, wholly without local influence, herself deemed a lunatic by many. That person is Dorothea L. Dix, who, by the aid of a big heart and a high purpose, has stood before Senators, Presidents, Congresses, and Crowned Heads, the earnest advocate of suffering humanity. And powerless in purse, person, position, in all, but an absorbing and consuming love for the afflicted of her race, she has caused light-houses to be raised, and life-boats to be established along the American coast, and lunatic asylums to be built and endowed in nineteen States of the Union; and, not satisfied with all this, has crossed the seas, and ferreted out facts in the wilds of Scotland, whose bare recital has thrilled the House of Commons, and set a nation to work—a nation! the proudest and strongest of the earth!

The lesson of this recital is tenfold. By how much happier the reader's condition is, than that of the helpless, hopeless, lunatic, and his sister, let him be daily, hourly thankful!

Then inquire what you have done? what are you doing? what ought you to do? what could you do, if you were to try, and had a heart in it, for the poor and wretched of your kind!

